# St. Bartholomew's Hospital

**Published:** 1904-01-30

**Authors:** 


### HOSPITAL ADMINISTRATION.

### CONSTRUCTION AND ECONOMICS.

### ST. BARTHOLOMEW'S HOSPITAL.

Sir William Hart Dyke struck the true note when he pointed out that, however unanimous the meeting at the Mansion House, consisting of friends of Bart's might be, it was unfortunately true that there was outside that meeting, in several influential quarters, strong opposition to the proposals submitted to that meeting. Sir William Hart Dyke is perfectly correct, and we believe the opposition to be due largely to the want of a definite policy carefully thought out and precisely defined by the Governors of St. Bartholomew's Hospital. The best interests of St. Bartholomew's Hospital, and indeed of the hospitals of London, demand, that the present *impasse*, for it is an *impasse* within and without so far as Bart's is concerned, shall be removed without delay. The speech of the treasurer, who put the new wards last on a long list of building requirements, and of Sir William Church, who dwelt with loving affection upon the efficiency of the existing ward blocks, coupled with the list of contributors read by Sir William Treloar, and the relatively small sum given by some of the most wealthy names on the list, lead to the conclusion, that the authorities of Bartholomew's do not believe in the success of their own appeal, and are prepared to let the matter drag on indefinitely year after year.

It is a public duty, therefore, to press the treasurer for a clear statement on these points, for otherwise we foresee that the existing site may be spoilt for modern hospital purposes. This may be easily done by erecting an out-patient department upon a portion of the site which will render it impossible for an entirely new hospital to be ever erected thereon upon the best plan, which would safeguard the comforts of the patients, provide for the maximum of economy in administration, and secure the fullest hygienic conditions of which the site is capable. The Mansion House meeting was ostensibly a meeting of citizens, and it is a matter for regret that it was not so treated by those responsible for its management. It is always bad policy to refuse, at such a meeting to permit anybody to speak, except those included in the official programme, and we regret, in the interests of Bart's, that the questions put by Mr. Hastie were not precisely answered. The omission to answer them indicates in our view the inside position of Bart's, and shows that the controlling authorities are not really familiar with the many-sided problem presented for solution. To illustrate this we give the questions and answers.

Mr. Hastie's Questions.

1. Whether it could be definitely stated that the sale of the site and removal of the hospital would still necessitate an appeal ?

*Answer.*—If the figures of those who advocate the removal to a new site are to be relied upon, and the value of the land is what the surveyors of the hospital put it down at, removal would necessitate an appeal to the public.

2. Whether the hospital could not do its work equally well on another site ?

*Answer.*—It could not do so, because it is desirable to have one general hospital in the City of London. Its removal to another district would deprive thousands of poor people of the benefits they receive from Bart's to-day. It would also be opposed to national and Imperial interests, for it would cripple or destroy the medical school.

3. Whether the majority of the patients come from the vicinity of Smithfield or within one mile radius of it ?

*Answer.*—The answer to this must depend first upon the meaning attached to the word vicinity. The Mansion House and the Governors with the medical staff have in effect answered this question in the affirmative. Secondly, there is no means of ascertaining how many patients come from within the mile radius.

4. Whether the Governors felt themselves tied by the decision of the Mansion House Committee ?

*Answer.*—The Governors have unanimously decided, as a matter of business, to adopt the decision of the Mansion House Committee as to the site.

5. Whether the architect of the hospital would give his assurance that a hospital could be erected on the Smithfield site in accordance with the requirements of modern science ?

*Answer.*—The Model Pavilion plan which we published last week proves that the new hospital can be so erected in accordance with the requirements of modern science, provided that the Nurses' Home and Medical College are removed to a new site.

We desire that Bart's shall have an entirely new hospital upon its present site and that that hospital shall be erected forthwith. It is to be regretted therefore that the questions of Mr. Hastie were not answered, as we have shown they might perfectly well have been, at the Mansion House meeting. We are afraid that the treasurer and his immediate supporters do not realise, that the way to get the money is to set about he matter in a perfectly frank and businesslike spirit, and to so organise their forces as to secure, that the whole £500,000 shall be raised within twelve months from October next. This can be done if the proper means are adopted to accomplish it. At present there is no Appeal Committee, no Building Committee, and no perfected plan on which to base such an appeal, and until these are forthcoming, in our judgment, it is suicidal folly to attempt to raise so large a sum of money. Indeed the public and the press declare correctly, that if the attempt is persisted in at the moment it may mean a set-back for St. Bartholomew's Hospital which it will be very difficult, if not impossible, for that institution to overcome.

This is the precise fact, which everybody who is familiar with the business of raising money, and a large number of the best friends and well-wishers of St. Bartholomew's Hospital fully realise. It will be a thousand pities, therefore, if the truth is not grasped by those immediately responsible for the conduct of the affairs of St. Bartholomew's Hospital, as matters stand at present our oldest hospital is threatened with very grievous disaster.

### CROWDED MEETING AT THE MANSION HOUSE.

### APPEAL TO THE CITIZENS FOR A REBUILDING FUND.

THE LORD MAYOR, presided at a meeting at the Mansion House on the 26th inst., convened by his Lordship and Sir Trevor Lawrence, the treasurer of St. Bartholomew's Hospital, in furtherance of the appeal by that hospital for funds for the erection of new buildings.

The Egyptian Hall, in which the meeting took place, was crowded, close upon a thousand persons being present, while the platform was filled with gentlemen representative of the City and of the hospital.

The Lord Mayor said they had been summoned there that day by the Governors of that splendid institution, St. Bartholomew's Hospital, to obtain their support and assistance in the scheme of rebuilding the hospital which the Governors were about to carry out. He thought it would best meet their convenience if, without further remarks, he asked the treasurer of the hospital to make a statement in relation to the scheme.

Sir Trevor Lawrence at the outset craved the indulgence and kindness of his hearers on that important occasion. He said it was quite impossible for anyone occupying the position he did to fail to feel the great burden and responsibility which rested upon his shoulders. He was bound to say that he thought someone more able than he to move the great meeting he saw before him should have been entrusted to make the statement. If ever there was a time when he envied the golden words of a great advocate, it was then. He thought, however, he could venture to say that he had some claim to speak, however inefficient his words might be, on behalf of that great hospital. He was educated there himself, and, as many of them knew, his father was a member of the staff for many years, and, moreover, he (the speaker) had been treasurer of the hospital now for nearly 12 years. It was impossible for anyone to occupy that position without feeling what a great and far-reaching institution he was connected with. This appeal was made with the sanction and sympathy of H.R.H. the Prince of Wales, the President of the hospital. (Hear, hear.) He held in his hand a letter which, by the direction of his Royal Highness, was written by Sir Arthur Bigge to him (the speaker), stating that as President of St. Bartholomew's Hospital, and in the special circumstances of the case his Royal Highness was pleased to associate himself with this appeal, which had his Royal Highness's sanction and sympathy. (Applause.) A little while ago he had the honour of meeting his Royal Highness, who they were anxious should take the chair at their meeting in the City. His Royal Highness, however, was not able to do so, for reasons which he could not dispute had great force. His Royal Highness stated that his first duty was towards the King's Hospital Fund, of which he was chairman, and he thought he could not go to the public and with one hand appeal on behalf of the King's Fund and with the other hand appeal for money on behalf of some particular hospital. Moreover, he was President of more than one hospital, and if he made a special appeal for one he would naturally be expected to make one for another when it desired to raise funds. There was one particular point to which he (the speaker) would like to refer, and that was the question of the site of the hospital. An influential daily journal had devoted a good deal of its space to impress upon the public that St. Bartholomew's should be removed from its present site. He thought that question had been thoroughly thrashed out by the Mansion House Committee. (Hear, hear.) That Committee consisted of 16 members—nine appointed by the ex-Lord Mayor, Sir Marcus Samuel, and six appointed by the Governors—those nominated by the Lord Mayor being independent persons who brought a perfectly independent and unbiassed mind to bear on the question. What was the result of that Committee's labour? After sitting for four days and hearing a good deal of evidence, the Committee practically unanimously decided in favour of the retention of the hospital on its present site. There was only one dissentient, and that was a gentleman who was known to have settled convictions on the other side. It was the opinion of the persons who came before the Committee to give evidence that it was necessary to maintain St. Bartholomew's Hospital on the site it had occupied for 800 years. St. Bartholomew's was one of the few remaining links which still existed between the City and William the Conqueror. It was a remarkable circumstance that a great charitable institution should have occupied the same site for 800 years. The witnesses who came before the Mansion House Committee were people of large experience. He was much struck by the evidence of Captain Nott Bower, the City Commissioner of Police, who said that of the accidents which occurred in the City 65 per cent. were taken to St. Bartholomew's Hospital. He would like to read an extract from an article which appeared in the *Times* on the 16th inst., which put the matter forcibly and far more clearly than he could. Referring to alternative schemes that had been put forward the writer said "they involved either the complete or the partial removal of the hospital, and were met with an abundance of evidence that the present central position is practically the best that can be suggested, with reference either to the wants of London itself or to those of the much wider circle for which the hospital almost equally provides. The maintenance without shrinkage of the maternity department, strongly contrasting with the diminution of the same department at King's College, shows that the square mile around St. Bartholomew's still contains a large resident poor population, and in all London, perhaps excepting that of the docks, there is probably no locality more fruitful of accidents than Smithfield. The vicinity of eight railway termini to which passengers in thousands come daily, not only from every part of the suburbs, but also from every part of the Midland and Northern counties, is an equally important consideration. As with Rome, so all roads lead to St. Bartholomew's, and its removal, even to a suburb, would place serious difficulties in the way not only of the thousands of patients who now flock to it from all parts of the kingdom, but also in the way of the many poor who now visit their relatives within the wards, and to whom omnibus or tram fares would frequently be prohibitive. The absence of these people would not be regretted by the doctors or nurses, but would, sometimes at least, be a serious trouble to the sick."

Then in the *Times* of the 23rd appeared a letter from the staff of the hospital, which among other matters dealt with the question of situation of the hospital. "Its great accessibility," said the writers, "is a matter of the very highest importance to the poor of London. So long as the City is the heart of London and of the Empire, so long will access to it, and consequently to St. Bartholomew's, be cheap and easy, and so long will this charity remain a great consulting centre for the sick poor. The fact that its out-patients number about 150,000 in a year is a sufficient proof in itself of the usefulness of the hospital on its present site. There is no other district in any part of this city or in its suburbs where the hospital could be more advantageously placed, and none where a more healthy site could be found. But the situation of St. Bartholomew's is further of importance to its medical school, and we may be pardoned for thinking that this is also a matter of material public interest."

Sir Trevor Lawrence, continuing, said he noticed in the paper he had before referred to an article advocating the divison of the hospital into two parts—one part for more serious cases and another part for cases that were more or less convalescent. He thought this was one of the most undesirable suggestions that could have been made. (Hear, hear.) It would disturb the medical school, and be detrimental to the welfare of the patients. It would be a disgrace to jeopardise the maintenance of that great school which had brought forth a long series of distinguished men from the days of Harvey down to the present time. (Applause.) He ventured to think that the value of the hospital site had been seriously and largely exaggerated. They had had the opinion of some of the most prominent land valuers in the City of London, and the valuers agreed and put the value of the site at far less than the newspaper he had referred to. The site of St. Bart's was compared with the prices obtained for the site of Christ's Hospital But the circumstances were different. In the case of Christ's Hospital two people wished to buy — the General Post Office, who eventually purchased one piece, and St. Bart's, who bought the other. If they were to move the hospital the Governors would be in the position of compulsory buyers and compulsory sellers, and everyone knew how unsatisfactory that must be. (Hear, hear.) There was another consideration which ought to weigh very considerably. On the present site they had a great general hospital—perhaps the most valuable site on which they could have a hospital—and surely they were not going to put monetary considerations before a great work of mercy and charity. (Applause ) With regard to having a branch hospital away from the present site, there was this consideration, that their patients were the poorest of the poor—people to whom the smallest sum was of the greatest consideration. Many of their patients were people paid by the hour, and if they had to spend their time in going to a distant hospital it meant so much less would be available on which to feed their families and themselves. To show the work the hospital did on the present site he mentioned that seven million patients had been treated in the last fifty years. He also had the figures for the first three weeks of this year and he found that in that period 15,247 attendances of patients had taken place. (Applause ) He dared say it had been noticed that one gentleman had pointed out that according to the census the populace was moving away from the hospital. He would remind that gentleman that the census was taken on a Sunday night and that everybody who could move away from the City on a Sunday night did so. If the census had been taken on a Monday morning the result would have been totally different. Patients went to see their doctors in the day time; not at night. People coming into the City to work had always at hand any medical assistance they might require. He did not think he need elaborate that subject any further; it would be admitted that these considerations showed conclusively that a hospital was required on the site where St. Bartholomew's stood, and that a great mistake would be made if they moved the hospital elsewhere. (Applause.) The next point was as to what the hospital required. The present buildings were erected about 150 years ago—though there had been many additions since. Although the wards were still able to show as good results as any in London, yet they were somewhat antiquated, and there were parts of the hospital which required immediate attention. They wanted a new out-patient department, and accommodation for an ophthalmic, an orthopædic, a skin, throat, ear, dental, electrical, and gynæcological departments. These departments ought to have separate accommodation because they required the use of complicated and delicate apparatus, and it was necessary that each department should have its own rooms so that the apparatus should not be liable to disturbance and injury by unnecessary movement. Then they wanted a new nurses' home—(applause)—and everyone who had ever been brought in contact with the nurses would sympathise with that. He thought the loyalty of these devoted women who served them in that capacity was one of which words failed to adequately express their appreciation. (Applause.) They prided themselves that all their nurses were ladies of good education and in many cases of good family, and he was bound to say from what he had seen that it was impossible to do justice to the work they did. They wanted a home for 240 nurses and 80 female servants. The nurses, as they knew, performed all the work connected with the patients, but it was not desirable that they should be asked to scrub the floors. They also wanted a new pathological department. One thing he ventured to express an opinion about was this: those were the departments on which the march of medical science in the future would depend; the future of medical science belonged to the scientific mind, and where progress was made it would be in that direction. Then they wanted a house for the vicar, the matron, the steward, and the clerk of the works; new boiler houses, disinfectant chambers, and new operating theatres. One member of the hospital staff told him the other day that he had a case which he was unable to deal owing to the fact that the operating theatres were so constantly occupied. Then they wanted subways because they often required to move patients from block to block, and it was necessary that this should be done without having to take the patient into the open air. They also wanted lodge gates, an isolation block, and finally blocks of new wards six in number. He was giving the details on this subject so far as they had been arrived at. Before the hospital undertook the matter very careful consideration would be given to all the details. The total cost was estimated at £438,000, and that would provide an absolutely new hospital. (Hear, hear.) It was the intention of the hospital Governors, in conjunction with the medical staff, to secure an absolutely and entirely new hospital. (Applause.) In this matter they would have the advice of a good and competent building committee. He had been asked by a gentleman for whom he had the greatest respect, and who was present, whether it was not possible to place the names of that Committee before the meeting, but the selection of a good building committee was a matter of time, because when they had selected a gentleman they were not always sure whether he would be able to serve. With regard to income and expenditure, the average income of the last five years was £68,000 per annum. He referred to this specially because they had been told repeatedly that the income of the hospital was £100,000 a year. The average for the last ten years was £67,000 per annum. The average expenditure for the last five or ten years—it made little difference which—had been £67,000, so that there had been a small surplus, and that surplus had been applied towards the purchase of the land from Christ's Hospital. Owing to that purchase they would in future be £6,000 short of the average expenditure of the hospital. In conclusion he thanked the ladies and gentlemen for their presence at the meeting. He could assure them that he felt the responsibility of this appeal and could have wished that it had met with greater general acceptance. There was no nobler institution in the world than St. Bartholomew's Hospital. (Applause.) Anyone who walked through the wards of the hospital and saw that great work of alleviating suffering and restoration to health must feel his heart moved—if he had a heart to move. He could not believe that this great City would be deaf to the cause of duty, but would rally to the support of St. Bartholomew's. He could not think that that great City, which could always find money for everybody, would fail in its duty to place that institution, the oldest of its kind in the kingdom and probably in the world, in the position which it should occupy. (Applause.)

The Bishop of London moved the first resolution:—

That this meeting, having heard the statement of the treasurer of St. Bartholomew's Hospital, cordially approves of the decision of the Governors to reconstruct the hospital on its ancient City site, extended as it has recently been by the addition of one and a-half acres.

He said it gave him great pleasure to move that resolution as Bishop of London, because by trying to care for the bodies as well as the souls of the people in London he was carrying on the traditions of his predecessors. It was a Prebendary of St. Paul's who founded the hospital and it was a Bishop of London who procured the land upon which it was built. He reminded the Lord Mayor that their predecessors in office had long ago been associated in support of this noble institution. This was far the oldest hospital in the United Kingdom. As he got on in life he looked more and more with reverence on pioneers and he was now appealing for the pioneer hospital of the country. (Applause.) The second reason for supporting the appeal was the splendid work that this hospital had done for poor humanity in the last 800 years. (Applause.) As many of them knew, he had spent nine years of his life in the slums of London, and it was only those who had spent years in the slums who could realise what these hospitals were to the poor. When he spoke of one hospital he spoke of them all. He loved the London, the great hospital of the East End, he loved King's College Hospital, and was going to help its chairman in an appeal for money in order to move to South London. There was no rivalry between hospitals, and he thought the heart of London ought to be big enough for all. They must not forget the splendid work St. Bart's had done throughout the world by means of its medical school. A Bart's man was a marked man throughout the world for the splendid education he had received. He had often visited the hospital when the staff did not know he was there, looking after one of his patients, and he knew how excellently the hospital was worked. It was not his business to mix himself up with the question of sites, but he was quite content to take the verdict of the sixteen honest men who had studied the question with an open mind. (Applause.) He took it from them that it was best to build on the present site. The argument as to the value of the site might be carried too far. He could imagine a very plausible argument being made for the removal of St. Paul's Cathedral. (Laughter and applause.) Naturally that was a suggestion which he for one should strongly oppose. They must have some churches in the City. The removal of King's College Hospital to South London made the retention of Bart's all the more necessary. He took it from the report of the Mansion House Committee that the hospital had come out with flying colours, and they wanted St. Bartholomew's to be the best hospital that could be turned out for the City of London. He stood there that day to plead very earnestly for the unfathomable generosity of the City of London to open its hand once more and make St. Bartholomew's the best hospital they could. He had great pleasure in moving his resolution.

Sir William Church, K.C.B., President of the Royal College of Physicians and consulting physician to the hospital, seconded the motion. He said when he remembered the long connection he had had with St. Bart's, and the 40 years he had been in its wards, he thought he ought to know something of the work it did, and also its present needs. During those 40 years many improvements had been made, but no further improvements were now possible to facilitate the ever-increasing work. He thought he could confidently appeal to the City of London to help to remove the difficulties under which they had so long laboured. He believed certain objections had been made to their coming before them that day because there were no detailed plans of the rebuilding of the hospital to put before them. He could not believe these objections were seriously advanced, because they might be sure that the reconstruction committee, in providing at once the buildings so urgently required would do nothing to hamper the work of the hospital. As to the need for the hospital in the heart of the City he need only remind them that they were in the closest proximity to the daily influx of between 30,000 and 40,000 workers. Cases of accident and sudden sickness arose during the working hours of the day. There was an impression abroad from a perfectly unauthorised statement which had appeared in the press that the present wards of the hospital were so out of date that they were detrimental to the health of the patients in them. That might be dismissed from their minds entirely. He might say that the results which had been obtained by their medical staff were as favourable as could be shown by the most up-to-date hospital.

Mr. John Tweedy, President of the Royal College of Surgeons, supported the motion, and said that he did so the more gladly that he was not a Bart's man. Having read with an open mind the careful report of the Mansion House Committee and the conclusions at which they arrived, and having heard the various arguments reinforced in so telling a manner by the Bishop of London, he had no hesitation in saying that the removal of the hospital would be almost a calamity, and at all events a disgrace to the City.

Dr. Gee also supported the resolution. He said if the great age of St. Bartholomew's meant decrepitude and decay it would be very sad, but it was never more full of life and energy than at the present moment. Growth was a sign of youth, but the hospital had grown so fast that it had outgrown the buildings erected in the eighteenth century. Let them inscribe on their escutcheon, "Here I am, and here I mean to stay."

The motion was then put to the meeting and carried.

The Hon. Alban Gibbs, M.P., proposed the second resolution—

That this meeting pledges itself to support the Governors of St. Bartholomew's Hospital in their appeal for the funds necessary for the reconstruction of the hospital, and earnestly commends to the generosity of the public this—the first appeal which the hospital has made for 150 years.

He said he only proposed to speak very shortly because the resolution was merely consequential, and he considered they had passed it already when they decided not to move. Having settled that they necessarily settled that they had got to pay for it. Everybody knew that hospitals did not grow, and that money was required to get them built.

The Master of the Mercers' Company seconded the resolution.

Dr. Adler, the Chief Rabbi, supported the resolution. He said that having been a visitor to the hospital, he could testify to the great kindness and consideration with which his own poor had been tended in the wards of St. Bartholomew's, and therefore he hoped that the members of his own community would respond with whole-hearted generosity to the appeal which had now been promulgated. (Applause.)

Sir William Hart Dyke, M.P., also supported the resolution. He said he was one of the extreme outsiders called to the committee which dealt with this question. Although that meeting might be unanimous the outside public and many of those to whom they had to appeal were not unanimous, and therefore it was for that reason that he asked their attention. He went on the committee without any bias whatever—without even the luxury of a fad or crotchet. After hearing the evidence brought before the committee, he came to the conclusion that the appeal which was being made was a just and right appeal. The evidence was overwhelming in favour of the hospital being retained on the present site. (Applause.)

Mr. A. Bowlby, C.M.G., F.R.C.S., also supported the resolution. He said he was there to emphasise the fact that the staff of Bart's was entirely unanimous in their support of these proposals, and he was able to say on behalf of his colleagues that they were absolutely unanimous as to the necessity of having certain buildings and the erection of a new hospital. If they did this he ventured to say that the success of the hospital would be greater than in any of the 800 years it had been in existence and they might fairly hope to rival the work of their predecessors.

Mr. Hastie, speaking from the body of the hall, put a series of questions whether the sale of the site would not avoid the necessity for an appeal; whether the greater part of the patients came from within a mile radius of Smithfield; was it impossible to carry out the work anywhere else; and did the authorities consider themselves absolutely tied down by the report of the Mansion House Committee?

Sir William Treloar replied. He said the site of the hospital had been called a gold mine, but in his experience land had often been called a gold mine which had not turned out so. He was not prepared to answer question No. 2; he did not see it had the slightest bearing on this appeal. In any case he declared the committee meant to prosecute the appeal and meant to get the money. (Loud applause.) They were sure they knew something about the City in which they had lived for many years, and they intended to get the money.

Another gentleman in the body of the hall essayed to speak, but the Lord Mayor refused to allow him to proceed, saying that he could vote against the resolution if he liked.

The resolution was then put, three hands being held up against it.

Sir William Treloar then read the list of subscriptions, which, he said, already amounted to £40,000. The chief contributors were:—Her Majesty the Queen, £1,000; the Merchant Taylors' Company, £2,500; Mr. Ebenezer Holman, £2,100; Mr. Casey Lovell, £2,000; the Goldsmiths' Company, £2,000; Sir Trevor Lawrence, £1,000; Mr. A. Lawrence, £1,000; Mr. Cohen, M.P., £1,000; Mr. T. Lawrence, £1,000; Messrs. J. Henry Schroeder and Co., £1,000; the Mercers' Company, £1,000; Mr. H. Perrin, £1,000; Messrs. Speyer, £1,000; Sir Reginald Hanson, £1,000; Messrs. Hill, £1,000; and Messrs. Rothschild, 600 guineas. Sir William Treloar added that he had received £1,500 from members of the Corporation and hoped to get much more.

The Master of the Merchant Taylors' Company briefly moved a vote of thanks to the Lord Mayor for presiding and for granting the use of the Mansion House.

The Rev. J. R. Campbell, M.A., seconded, and the motion, which was supported by Mr. J. Langton, F.R.C.S., was carried unanimously.

The Lord Mayor acknowledged the vote and the proceedings then terminated.

